# “Patient Comfort Can Be Sacrificed for Patient Safety”—Perception and Practice Reported by Critical Care Nurses Toward Physical Restraints: A Qualitative Descriptive Study

**DOI:** 10.3389/fmed.2021.573601

**Published:** 2021-07-23

**Authors:** Nianqi Cui, Ruolin Qiu, Yuping Zhang, Jingfen Jin

**Affiliations:** ^1^Department of Nursing, The Second Affiliated Hospital Zhejiang University School of Medicine (SAHZU), Hangzhou, China; ^2^Faculty of Nursing, Zhejiang University School of Medicine, Hangzhou, China; ^3^Changxing Branch Hospital of SAHZU, Huzhou, China

**Keywords:** physical restraints, critical care nursing, perception, practice, patient safety, patient comfort, qualitative research, thematic analysis

## Abstract

**Aim:** The aim of the study was to explore the perception and practice of physical restraints used by critical care nurses.

**Design:** A qualitative descriptive design was used.

**Method:** From December 2019 to May 2020, a one-to-one, semi-structured in-depth interview with 10 critical care nurses from two intensive care units in a tertiary general hospital with 3,200 beds in China was conducted using the method of purposeful sampling. The data were analyzed using inductive thematic analysis.

**Findings:** The perception of physical restraints among critical care nurses was that patient comfort can be sacrificed for patient safety. Physical restraints protected patient safety by preventing patients from unplanned extubation but influenced patient comfort. Physical restraints were common practice of critical care nurses. Relative physical restraints provided patients with more freedom of movement and rationalization of physical restraints which were the practical strategies.

**Conclusion:** The study identified problems in critical care nurses' perception and practice on physical restraints. Critical care nurses are confident that physical restraints can protect patient safety, and the influence of physical restraints on patient comfort is just like the side effect. Although physical restraints were common practice, critical care nurses still faced dilemmas in the implementation of physical restraints. Relative physical restraints and rationalization of physical restraints help critical care nurses cope with the “bad feelings,” which may also be the cause of unplanned extubation. It is necessary for the adaptation of clinical practice guidelines about physical restraints for critically ill patients in the Chinese context, to change the perception and practice of critical care nurses and deliver safe and high-quality patient care.

## Introduction

Patient safety has been a global health priority, while 134 million adverse events occur each year due to unsafe care in hospitals in low- and middle-income countries, contributing to 2.6 million deaths annually (1). The delivery of safe, high-quality patient care is of utmost importance to nurses. As nursing care spans all areas of care delivery, nurses are well-placed to prevent harm to patients and improve the quality and safety of healthcare delivered across all settings. As such, nurses should be central to the design and operation of all health providers' patient safety systems and processes (2).

Unplanned extubation (UE) is a serious adverse event that threatens the safety of critically ill patients because it is associated with a higher risk for mortality, morbidity, and resource utilization (3). Prevention of UE has also been the reason for the use of physical restraints (PR) by critical care nurses, although there is no evidence to establish that it is effectual and it is also associated with a higher risk for mortality and morbidity. In light of this, what are the perception and practice of critical care nurses toward PR? When we understand the perception and practice in which critical care nurses use PR on patients, we will provide more targeted measures to reduce the rate of PR on critically ill patients, thereby delivering safe, high-quality patient care.

### Background

Critically ill patients admitted to intensive care units (ICU) often need more invasive operations (e.g., mechanical ventilation and hemodialysis) due to the needs from their condition (4), which can also lead to acute pain, discomfort, sleep deprivation, agitation, and delirium (5). Agitation, for example, can propel patients to resist the ventilator, thus increasing the oxygen consumption, causing them to accidentally remove various devices and catheters on them and even posing life-threatening risks (6). Therefore, the main reason for the use of PR around ICU is to prevent patients from accidentally removing the catheters or devices needed to protect their safety (7).

The definition of PR is “Any action or procedure that prevents a person's free body movement to a position of choice and/or normal access to his/her body by the use of any method, attached or adjacent to a person's body that he/she cannot control or remove easily” (8). Although PR was used to prevent UE, there were many studies that proved PR is one of the risk factors that account for UE and cannot protect patient safety (6, 9). Indeed, its use has been proven to cause pressure injuries (10) and worsen agitation (11), delirium (12), and neurovascular complications (13). However, PR is widely used in ICU around the world (14).

Many institutions have suggested that the use of PR should be reduced. For example, the Government of Ontario released the Patient Restraints Minimization Act in 2001 to “minimize the use of restraints on patients and to encourage hospitals and facilities to use alternative methods, whenever possible, when it is necessary to prevent serious bodily harm by a patient to himself or herself or to others” (15). Just like nurses at a nursing home, psychiatric, general medical-surgical unit, critical care nurses also play a key role in practice processes about PR (14). Therefore, it is very important to identify the perception and practice of critical care nurses for the reduction of PR.

The Registered Nurses Association of Ontario (RNAO) issued clinical practice guidelines on the alternatives to PR in February 2012, aiming to help nurses reduce the use of PR, or use it in a more reasonable and standardized way, and to provide effective alternatives of PR (16). The clinical practice guidelines (CPGs) are a convenient way of packaging evidence and presenting recommendations to healthcare decision-makers (17). However, the development and updating of high-quality CPGs require substantial time, expertise, and resources (18). Guideline adaptation is the systematic approach to the endorsement and/or modification of a guideline(s) produced in one cultural and organizational setting for application in a different context. Where high-quality guidelines are already available, adaptation may be used as an alternative to *de novo* guideline development to customize the existing guideline to the needs of local users (19). There are currently no CPGs on PR in China, while other countries had, so we hope to be able to adapt existing guidelines to apply in the Chinese context (20). We use the CAN-IMPLEMENT approach to adapt the guidelines (21). According to methodological requirements, the first phase is to identify the problem/issue, and this study is the first step in our guideline adaptation.

## Method

### Aim

The aim of the study was to explore the perception and practice of PR used by critical care nurses.

### Design

A qualitative descriptive design was used (22), adopting methods from Patton (23). The choice of qualitative descriptive study is determined by the aim of our study since qualitative descriptive studies tend to provide the most direct and essential answers to the concerns of practitioners or policymakers (24).

### Participants

The sampling strategy combined maximum variation sampling with criterion sampling (23). The criteria for the inclusion and exclusion of participants are shown in Supplementary Table 1. For participants who meet the criteria, they were selected after taking into consideration the representativeness of such factors as gender, age, highest academic qualification, title, and years of experience in critical care. The sampling process took place from December 2019 to January 2020 at two ICUs in a tertiary general hospital with a total of 3,200 beds in Hangzhou, China.

### Data Collection

Data collection was actually implemented from April to May in 2020 since the original schedule beginning in February 2020 was delayed due to the impact of the COVID-19 pandemic. The data were collected through a one-to-one, semi-structured in-depth interview, which was conducted by a male nursing doctoral student (N.C) who had experience with qualitative research (25–27). The interview guide was developed adopting methods from Kallio et al. (28), (i) identifying the prerequisites to use a semi-structured interview; (ii) retrieving and utilizing the previous knowledge; (iii) formulating the preliminary interview guide; (iv) pilot testing; and (v) presenting the complete interview guide. Before the pilot testing, the interview guide was reviewed by a qualitative researcher (J.Z) (Supplementary Table 2). The interview took place in a quiet and private office outside the medical area and wholly recorded. The interview was not unfolded strictly in accordance with the interview guide but followed the thoughts of the participants, and the interview spanned 50–70 min. Data collection occurred concurrently with data analysis. This process helped researchers identify thematic saturation, which occurred with the eighth critical care nurse. To confirm thematic saturation, two additional critical care nurses were interviewed. However, these additional interviews did not bring out new themes. Hence, the recruitment of participants continued until no new thematic emerged.

### Ethical Considerations

Ethical approval was obtained from The Second Affiliated Hospital Zhejiang University School of Medicine (SAHZU, No. 2020131) before the onset of the study. Ahead of these interviews, written informed consent was provided by each participant who agreed to participate. All participants could withdraw from the study at any stage without needing to disclose the reason. The audio recordings, as well as transcripts, were tagged with numbers so that confidentiality and anonymity were assured and were safely archived in files protected by passwords.

### Data Analysis

The data were analyzed in the framework of thematic analysis (29) because it can construct themes related to the research questions through the analysis of the data. Thematic analysis, with an inductive approach, was undertaken by two researchers (N.C and R.Q) using Braun and Clarke's six-step framework: (i) become familiar with the data; (ii) generate initial codes; (iii) search for themes; (iv) review themes; (v) define and name themes; and (vi) write up the final report. The transcription of the research materials was carried out independently by the researchers and was also cross-examined to make sure that the contents of the transcription were correct. An open coding process was used, so codes were not set but developed and modified during the coding process. The two researchers jointly reviewed the themes they had extracted independently and exchanged opinions with each other. The ultimate theme was then determined together with the rest of the authors through the consensus process. Examples of quotations and themes/subthemes are shown in Supplementary Table 3.

#### Rigor

The role of the interviewer was assumed by N.C, a researcher trained in qualitative interviews, who had 4 months of clinical practice experience in ICU in a non-study site, and whose research direction during the master's degree was PR on critically ill patients. Therefore, he was familiar with this field of study. There had been no contact between him and these participants before. The participants were sampled through the maximum variation sampling with criterion sampling, which is believed to be able to improve the representation of the participants. The preliminary interview guide was reviewed and revised by a qualitative researcher before conducting the formal interview. Prior to the analysis of the data, the two researchers have trained again about thematic analysis, and the thematic saturation ended when both researchers agreed. We applied the Standards for Reporting Qualitative Research as the guideline to make sure the reporting of the study was transparent (Supplementary Table 4) (30).

## Result

### Participant Characteristics

Ten participant characteristics are found in Table 1.

**Table 1:** Participants' characteristics.

**Characteristics**	**No. (*n* = 10)**
**Gender**	
Male	3
Female	7
**Age (years)**	
25–30	5
31–35	3
36–40	1
>40	1
**Highest academic qualification**	
Master degree	2
Bachelor degree	8
**Title**	
Intermediate title	5
Junior title	5
**Years of experience in critical care**	
1–5	3
6–10	4
11–15	2
>15	1
**Position**	
Nurse team leader	4
Registered nurse	6
**ICU specialist nurse or not**	
Yes	2
No	8
**Type of ICU**	
General	6
Emergency	4

### Themes and Subthemes Constructed

**Figure 1:**
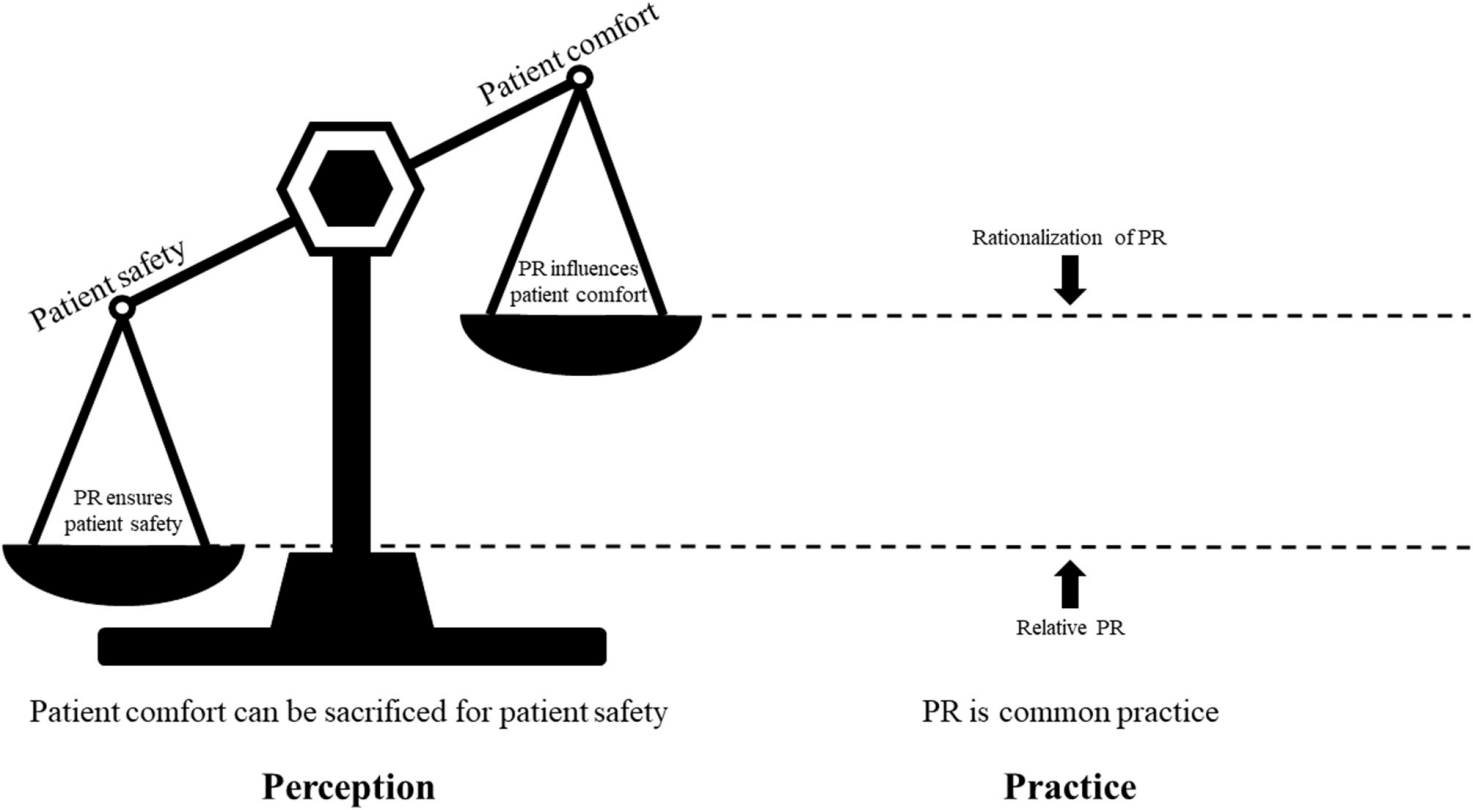
Relationship between themes and subthemes. Nurses consider that patient safety is more important, leading to common practice of physical restraints. Relative physical restraints and rationalization of physical restraints become strategies to cope with imbalance between patient safety and patient comfort. PR, physical restraints.

A total of two themes and four subthemes were identified as in Table 2. Figure 1 illustrates the relationship between themes and subthemes.

**Table 2:** Summary of themes and subthemes.

Themes	Patient comfort can be sacrificed for patient safety	PR is common practice
Subthemes	PR ensures patient safety	Relative PR
	PR influences patient comfort	Rationalization of PR

*PR, physical restraints*.

#### Patient Comfort Can Be Sacrificed for Patient Safety

Critical care nurses reported in the management of critically ill patients that patient safety is the primary goal. Like many treatments, there may be side effects while achieving the treatment effect. Many nursing interventions may affect the comfort of patients while ensuring the safety of patients. Fronting patient safety, patient comfort can be sacrificed. PR is an intervention of sacrificing patient comfort for patient safety. Nurse 2 stated,

*For nurses, our primary imperative is to ensure patient safety. Many procedures can cause even high degrees of discomfort for patients, such as sputum suction, but we need to do it for the patient's safety*.

Another participant expressed,

*I think it is acceptable to sacrifice patient comfort for patient safety. After all, patient safety is the most important. It's like patients experience nausea and vomiting following chemotherapy, but will we dissuade patients out of chemotherapy because of such side effects? (N-10)*

Although critical care nurses described that patient comfort could be sacrificed for patient safety, the ideal situation is to ensure patient safety without affecting patient comfort. Sacrificing patient comfort makes critical care nurses feel helpless and face a dilemma when implementing PR. Nurse 4 felt,

*When I implement PR on a patient, although I know he/she is very uncomfortable, but I have no other choice, I also feel helpless because I have no alternative*.

Another participant indicated,

*I will consider patient comfort, but I also need to consider patient safety. In fact, many clinical measures present such a dilemma. We must simply choose the lesser of two evils. (N-03)*

##### PR Ensures Patient Safety

Critical care nurses believed that patient safety means that patients are in stable conditions, avoiding harm. UE is an important adverse event threatening the safety of critically ill patients. When UE happened, the stable conditions will no longer exist immediately and patients will be in life-threatening conditions. Critical care nurses stated that ensuring UE does not occur in patients is an important embodiment of patient safety. Nurse 5 reported,

*If the patient is in a stable condition, then that means he/she is safe. Once UE occurs, the patients will turn quite green within a few minutes. […] UE can also cause blood pressure fluctuation and hemodynamic instability. These patients may require resuscitation. Some indwelling drainage tubes are implanted during surgical operations. If these tubes are removed, the patient may require reoperation. No matter how UE arises, the patient has been harmed by UE*.

Critical care nurses expressed that PR can prevent UE effectively and thus protect the safety of patients. In the view of critical care nurses, as long as patients can access tubes/catheters, there is a risk of UE. PR makes patients unable to access any tubes/catheters, thereby avoid UE. Nurse 9 said,

*PR is very effective in preventing UE. I had never encountered UE problems when a patient was restrained*.

Another participant described,

*No matter how you restrain patients, the core principle is that you can not let them access catheters or tubes. Once patients can access these tubes, UE becomes all but inevitable. (N-08)*

Critical care nurses expressed that, because light sedation has been suggested in ICU, patients who were in light sedation are easy to rouse and still at risk of UE. What is more, due to the unpredictability of UE, critical care nurses indicated that they could not prevent patients from UE even if they are at the bedside of the patients. Therefore, the existence of PR is necessary for patient safety. Nurse 2 said,

*Patients in the ICU frequently undergo procedures such as sputum suction and blood glucose measurement. When implementing such procedures on patients under light sedation, the patient could wake up suddenly. Without the implementation of PR, such patients are likely to extubate themselves*.

Another participant expressed:

*Even if I monitor the patient one-to-one, I can't prevent the patient from experiencing UE because the patient's self-extubation happens suddenly. When I realize self-extubation may be about to happen, it may already be too late for me to stop the extubation. (N-10)*

##### PR Influences Patient Comfort

Critical care nurses expressed that the most serious adverse effect of PR is about patient comfort. Patient comfort means that patients can move their bodies freely. Some specific movements of patients are based on physiological needs (e.g., relieving their itches). PR makes patients lose the freedom and cause discomfort. Nurse 1 stated,

*Critically ill patients lie in bed for a long time, and lying in the same position for an extended period of time is certainly uncomfortable, which requires postural changes. However, PR makes it impossible for patients to move freely. For instance, they cannot lift their arms. Therefore, they cannot scratch itches or wipe away their sweat. As a result, restrained patients often experience a high degree of physical discomfort*.

What is more, critical care nurses described that patient comfort is a holistic experience. The holistic experience of patients with PR in ICU will be very poor. They may think they are in jail or being abused. Some found it painful to think of experiences in the past and also painful to talk about them. Nurse 7 described,

*I think (patient) comfort refers to the holistic experience for patients, as in an experience without excruciating pain and suffering. Physical discomfort is secondary. The most important thing is that patient experiences with PR in the ICU can be terrible. When they transfer out of the ICU and recall this experience, they could feel that they were prisoners who were tied to the bed and abused, which could certainly cause mental trauma for patients*.

Critical care nurses' perception of the influence of PR on patient comfort comes from transposition thinking and communication with the discharged patients. Critical care nurses said that if they were patients, they would not want to be used PR. For instance:

*If I was a patient, I also wouldn't want to be restrained. (N-06)*

Another participant stated,

*Sometimes, I have occasionally met patients by chance who had been in ICU. When those patients talked about this (PR) that happened in the ICU, he/she all felt that experience was extremely painful. (N-09)*

#### PR Is Common Practice

At present, PR is still a routine measure in ICU. Some critical care nurses said that it does not need too much thinking for patients to implement PR, which is just a procedural measure. Nurse 6 stated,

*I don't have to think too much about implementing PR because it's a routine part of the ICU*.

Another participant said,

*PR is a routine measure that is directly implemented when patients come in. Then the nurse will decide whether to release it or not. (N-08)*

##### Relative PR

Critical care nurses indicated that there were two types of PR, positive PR and relative PR. Positive PR means that the nurse ties the restraint belt on the bedstead. The arm of the patient with positive PR is unable to move and lift. Positive PR is mainly implemented to patients with agitation, delirium, and uncooperation. Nurse 5 indicated,

*Patients experiencing agitation or delirium, and/or non-cooperative patients should be firmly restrained and not allowed to move at all*.

Relative PR means that the nurse ties the restraint belt on a movable object just like the bedside rail. The more cooperative and calmer patients are, the more likely they are to be with relative PR. The purpose of critical care nurses implementing relative PR is to provide more activity space for patients. Nurse 6 stated,

*...With calm patients, we will not restrain them too firmly. Generally, the restraint belt will be tied to the bed side-rails so that the patients will have more freedom of movement*.

Some critical care nurses said that patients with UE were the patients with relative PR. These patients may be with hypoactive delirium, and critical care nurses agreed that they mainly focused on hyperactive delirium patients and ignored hypoactive delirium patients. When patients had self-removal because of relative PR, it would lead to nurses being afraid of removing PR. Nurse 1 stated,

*We had thought that the patient was calm and cooperative and that the patient would not accidentally extubate themselves. Therefore, the restraints were quite loose, and we did not fasten the restraints to the bed frame in the hope that it would give the patient more freedom of movement. However, on many occasions, the patient's sudden movements pulled out the tubes. Following those incidents, the nurses no longer loosen or unfasten their patients' PR*.

Another participant said,

*In fact, the incidence of hypoactive delirium is very high, but we generally do not focus on this kind of patient. They are usually very calm, so generally, they won't be restrained very tightly. UE mainly maybe occur in these patients. We tend to pay more attention to patients with hyperactive delirium. (N-03)*

Critical care nurses admitted that relative PR might not be effective in preventing patients from UE. However, the implementation of relative PR can reduce their own pressure on patient safety and get a sense of security. Nurse 4 indicated,

*I know that relative PR may not be able to help (to prevent UE), but I am reassured when I see a patient with a restraint belt. If they are not restrained at all, I will be very stressed*.

##### Rationalization of PR

Although PR is a routine measure in ICU, critical care nurses still face dilemmas in implementing PR. When they did not know whether to implement PR or not, they would choose to implement PR. At this time, they would use the rationalization of PR. Rationalization of PR means that critical care nurses implement PR on patients by persuading themselves that it is for the sake of patient safety even if the patient may not need to be restrained. As long as the decision is for patient safety, it must be correct. Nurse 2 described,

*[…] When I am uncertain as to whether or not I should restrain a patient, I will implement PR, because it is for the patient's own safety*.

Another nurse said:

…* I'll tell myself that the decision to implement PR is correct because patient safety is the reason behind implementing PR. (N-05)*

Critical care nurses remarked that they felt sad in the face of patients suffering from PR, rationalization of PR is helpful to relieve their own sadness. Nurse 7 felt,

*I think most nurses feel bad when they see patients suffering because of PR. I feel better when I tell myself that PR is necessary and reasonable in order to protect patient safety*.

## Discussion

THE most important finding of this study was that the perception of PR among critical care nurses was that patient comfort could be sacrificed for patient safety (Figure 1). Patient safety was the primary imperative for the management of critically ill patients. The existence of PR was mainly to prevent UE, which was a serious adverse event threatening patient safety in their perception. Critical care nurses were confident that PR could prevent patients from UE and the influence of PR on patient comfort was just like the side effect of chemotherapy. Although PR was a routine measure, critical care nurses were still faced with dilemmas in the implementation of PR. Relative PR provided patients with more freedom of movement, but it might also be the cause of UE. Rationalization of PR might help critical care nurses cope with the “bad feelings” during PR practice.

A recent study explored the experiences of nurses using PR on mechanically ventilated patients in intensive care (31). In that study, critical care nurses perceived the importance of prioritizing and protecting patient airways over other aspects of patient care and airway protection was described as the most powerful driving force in their decision-making regarding PR application. These results were in accordance with our study indicating that patient safety is the primary goal and PR effectively prevents UE, thus protecting patient safety. This may well explain why critical care nurses described that patient comfort could be sacrificed for patient safety. In accordance with the present results, previous studies have demonstrated that critical care clinicians thought the restraints would help prevent UE and the consequences associated with UE (32). The most obvious finding to emerge from the analysis was that critical care nurses are convinced about the effectiveness of PR to prevent patients from UE. In their opinion, critically ill patients could easily be roused and woken because the current practice in sedation is light sedation and frequent stimulation.

However, this does not mean nurses do not care about patient comfort. The dilemma faced by nurses in the implementation of PR is good evidence. In our study, because the balance between patient safety and patient comfort could not be maintained, if the patient was not restrained, they would feel stressed because of patient safety. However, if they implemented PR on patients, they would be helpless and in bad feelings because of the impact of PR on patient comfort. This finding was also supported by Salehi et al. (33) that there were ethical dilemmas between the pressures to maintain patient safety and potential harm in PR implementation. The quantitative research also proved the existence of ethical dilemmas (34). It was reported by 76.5% of nurses that the ethical principle of non-maleficence was the most common ethical dilemma associated with PR application. Other ethical principles that were of concern for nurses around PR use were beneficence, respect of the individual, and autonomy.

Although the purpose of our study does not include the exploration of coping strategies for nurses facing ethical dilemmas in PR practice, two types in PR practice, relative PR and rationalization of PR, may be the coping strategies. PR limited the freedom of movement of patients. As a type of PR, relative PR reduced the pressure of critical care nurses on patient safety and gave them a sense of security. At the same time, relative PR was a measure with which nurses provide more freedom of movement as possible as they can, which contributed to patient comfort and helped to reduce the level of negative emotions of themselves. The standard of relative PR implemented by nurses was whether the patient is calm and cooperative. As critical care nurses worried, patients with hypoactive delirium might meet such criteria and be implemented with relative PR, and then UE occurred, which may also explain why critical care nurses think UE is unpredictable. What is worse, because patients had UE, nurses are not inclined to remove restraints in future PR practice, which might be the reason why PR is a routine measure. Our study also found that critical care nurses were not concerned about hypoactive delirium compared with hyperactive delirium. These results are in accordance with a recent study (35) indicating that healthcare staffs including nurses do not identify the symptoms of a possible hypoactive delirium.

Rationalization of PR was a practice for critical care nurses when they were faced with decision-making obstacles of PR. When critical care nurses were hesitant about implementing PR or not, they would choose to implement it because they considered that they have no alternative. By persuading themselves that the potentially wrong decision was correct, nurses could implement PR on patients. However, the PR implemented in this situation also caused negative emotions to themselves. When critical care nurses used the rationalization of PR, they would feel better. These results reflect those of Chuang and Huang (36) who also found that nurses kept telling themselves that PR use was humane and this decision was right when their feelings were contradictory. They forced themselves to believe that they were doing the right thing for the patients, even when they were not sure.

If health organizations hope to reduce the use of PR, the authors believe that the perception and practice of critical care nurses need to be changed. The literature (14) has shown that critical care nurses are often the staff responsible for deciding to initiate and remove PR. Therefore, their perception is crucial to the reduction of PR. Patient safety is the reason behind the implementation of PR. Nurses think that PR can prevent UE, so it can protect patient safety. It may be that not all UE have negative impacts on patients. UE may bring a positive outcome to patients, as in (6), which reported that patients with UE had significantly lower hospital mortality and hospital stay. What is more, studies have reported that PR are not effective at preventing UE (37). Results from a systematic review and meta-analysis showed that PR is a risk factor for UE (OR 3.10, 95% CI 2.21–4.34; *p* < 0.01) (38). In critical care nurses' perception, there is no alternative to prevent patients from UE except PR. However, the CPG released by RNAO, *Promoting safety: alternative approaches to the use of restraints*, focuses on alternative approaches for PR. The knowledge may help to change critical care nurses' perception of PR. In the authors' opinion, the reason why critical care nurses implement relative PR and rationalization of PR is their lack of the explicit decision-making tool for PR, which may alleviate some of the ethical dilemmas. As CPGs may be a convenient way of packaging evidence and presenting recommendations for critical care nurses' decision-making about PR, and high requirements of resource about the development of high-quality CPGs, it is necessary for the adaptation of CPGs about PR for critically ill patients in Chinese context. In the process of the CPG adaption for PR on critically ill patients, it is necessary to integrate the relevant guidelines about the management of PR and UE of patients with mechanical ventilation for critically ill patients, thereby helping critical care nurses clarify the criterion for implementing and removing PR and avoid the relative PR and rationalization of PR.

## Limitations

The first limitation of this study is about the participants. All of them are from a tertiary general hospital located in Hangzhou, China. We combined maximum variation sampling with criterion sampling and considered multiple factors in the selection of research subjects so that they were well-represented. However, the critical care nurses from other cultural and linguistic backgrounds are not adequately represented. At the same time, under the principle of informed consent, all subjects were critical care nurses who were willing to share their perception and practice on PR, while nurses who also have perception and practice on PR but were reluctant to share may have divergent opinions. The second limitation is about methods of data collection. Observational research methods can explain behavior and practice patterns in ways that an interview design cannot. We regret that our own resource, ability, and time limitations preclude us from using this method.

## Conclusion

Critical care nurses believed that patient comfort can be sacrificed for patient safety and they were confident that PR can protect patient safety and the influence of PR on patient comfort is just like the side effect. Although PR was commonly implemented by critical care nurses, they still faced with dilemmas in the implementation of PR. Relative PR provided patients with more freedom of movement, and rationalization of PR helped critical care nurses to cope with the “bad feelings,” which may all be the cause of UE. At present, there are still some problems in critical care nurses' perception and practice on PR, so it is necessary for the adaptation of CPGs about PR for critically ill patients in the Chinese context, so as to change the perception and practice of critical care nurses and deliver safe and high-quality patient care.

## Data Availability Statement

The datasets presented in this article are not readily available because The datasets generated and/or analyzed during the current study are not publicly available due to concerns about participant privacy. Although references to specific institutions have, to the best of our ability, been removed, there may be areas where participants have provided details in interviews about institutional characteristics that could be identified. Our participants did not consent to have full transcripts of their interviews made publicly available. Requests to access the datasets should be directed to zrjzkhl@zju.edu.cn.

## Ethics Statement

The studies involving human participants were reviewed and approved by Human Research Ethics Committee of The Second Affiliated Hospital Zhejiang University School of Medicine (No.2020131). The participants provided their written informed consent to participate in this study.

## Author Contributions

NC, RQ, YZ, and JJ made substantial contributions to conception and design, or acquisition of data, or analysis and interpretation of data, involved in drafting the manuscript or revising it critically for important intellectual content, gave the final approval of the revision to be published, each author has participated sufficiently in the work to take public responsibility for appropriate portions of the content, and agreed to be accountable for all aspects of the work in ensuring that questions related to the accuracy or integrity of any part if the work are appropriately investigated and resolved. All authors contributed to the article and approved the submitted version.

## Conflict of Interest

The authors declare that the research was conducted in the absence of any commercial or financial relationships that could be construed as a potential conflict of interest.

## Publisher's Note

All claims expressed in this article are solely those of the authors and do not necessarily represent those of their affiliated organizations, or those of the publisher, the editors and the reviewers. Any product that may be evaluated in this article, or claim that may be made by its manufacturer, is not guaranteed or endorsed by the publisher.
