# Supplementary material for: “Patient Comfort Can Be Sacrificed for Patient Safety”—Perception and Practice Reported by Critical Care Nurses Toward Physical Restraints: A Qualitative Descriptive Study
Source: Front Med (Lausanne). 2021 Jul 23;8:573601. doi: 10.3389/fmed.2021.573601 (PMC8342939; doi:10.3389/fmed.2021.573601)
Supplement: Supplementary file 1 [file Table_1.docx]

Supplementary Material

# Supplementary Table

Table.1 Inclusion and exclusion criteria

| **criteria** |
| --- |
| ***Inclusion criteria*** |
| Registered nurses in the study site |
| General intensive care units or emergency intensive care units |
| More than one year of experience in critical care |
| Experience of physical restraints within three months |
| Rotating work shift |
| ***Exclusion criteria*** |
| Registered nurses who were not on duty during the interview |
| Unable to consent to participate |

Table.2 Interview guide

| **Number** | **Questions** |
| --- | --- |
| 1 | Please detailed describe an experience of using physical restraints on patients. |
| 2 | What is your perception on physical restraints? |
| 3 | How you make the decision to use physical restraint on patients? |
| 4 | What are the factors that affect your decision-making? |
| 5 | Do you have anything else to add or share? |

Table.3 Themes, Sub-themes and Quotations of Perception and Practice Towards Physical Restraints

| **Themes** | **Subthemes** | **Quotations** |
| --- | --- | --- |
| Patient comfort can be sacrificed for patient safety | PR ensures patient safety | There is a value sequencing in the management of patients. The airway safety of patients is the most important, and patients need to endure discomfort. (N-01) |
|  |  | Although there is no clear data statistics, PR can effectively prevent UE. (N-07) |
|  |  | There is no more effective way to protect patient safety except for PR for hyperactive delirium patients without mechanical ventilation. (N-06) |
|  | PR influences patient comfort | When a patient wakes up and finds that he has an endotracheal tube in his mouth and his arms can't move, it must be very uncomfortable. (N-10) |
|  |  | If I remove PR of a patient, it is also for the patient comfort. (N-04) |
| PR is common practice | Relative PR | If the patient can cooperate, restraints will be loose. If he/she don't cooperate, restraints must be very tight. (N-09) |
|  |  | No matter how calm the patient is, they should be restrained slightly. In this way, I feel more at ease. (N-10) |
|  |  | UE occurred in this kind patients (with relative PR) will make me doubt my working ability. (N-07) |
|  | Rationalization of PR | Nurses will also give themselves psychological implications that PR is for the good of patients. (N-04) |
|  |  | It's really difficult to maintain the balance between patient safety and comfort, as long as PR protect patient safety, there is no problem. (N-01) |
|  |  | We all think about the patient, and so are the decisions about PR. (N-03) |

PR: physical restraints

UE: Unplanned extubation

Table.4 Standards for Reporting Qualitative Research (SRQR)

| **No.** | **Topic** | **Item** | **Details** |
| --- | --- | --- | --- |
|  | **Title and abstract** |  |  |
| S1 | Title | Concise description of the nature and topic of the study Identifying the study as qualitative or indicating the approach (e.g., ethnography, grounded theory) or data collection methods (e.g., interview, focus group) is recommended | √ |
| S2 | Abstract | Summary of key elements of the study using the abstract format of the intended publication; typically includes background, purpose, methods, results, and conclusions | √ |
|  | **Introduction** |  |  |
| S3 | Problem formulation | Description and significance of the problem/phenomenon studied; review of relevant theory and empirical work; problem statement | √ |
| S4 | Purpose or research question | Purpose of the study and specific objectives or questions | √ |
|  | **Methods** |  |  |
| S5 | Qualitative approach and research paradigm | Qualitative approach (e.g., ethnography, grounded theory, case study, phenomenology, narrative research) and guiding theory if appropriate; identifying the research paradigm (e.g., postpositivist, constructivist/ interpretivist) is also recommended; rationale^b^ | √  qualitative descriptive  naturalistic inquiry  relativism; subjectivism; |
| S6 | Researcher characteristics and reflexivity | Researchers’ characteristics that may influence the research, including personal attributes, qualifications/experience, relationship with participants, assumptions, and/or presuppositions; potential or actual interaction between researchers’ characteristics and the research questions, approach, methods, results, and/or transferability | √  2.6.1 rigour |
| S7 | Context | Setting/site and salient contextual factors; rationale^b^ | √ |
| S8 | Sampling strategy | How and why research participants, documents, or events were selected; criteria for deciding when no further sampling was necessary (e.g., sampling saturation); rationale^b^ | √  2.3 Participants  2.4 Data collection |
| S9 | Ethical issues pertaining to human subjects | Documentation of approval by an appropriate ethics review board and participant consent, or explanation for lack thereof; other confidentiality and data security issues | √  2.5 Ethical considerations |
| S10 | Date collection methods | Types of data collected; details of data collection procedures including (as appropriate) start and stop dates of data collection and analysis, iterative process, triangulation of sources/methods, and modification of procedures in response to evolving study findings; rationale^b^ | √  2.4 Data collection |
| S11 | Data collection instruments and technologies | Description of instruments (e.g., interview guides, questionnaires) and devices (e.g., audio recorders) used for data collection; if/how the instrument(s) changed over the course of the study | √  2.4 Data collection |
| S12 | Units of study | Number and relevant characteristics of participants, documents, or events included in the study; level of participation (could be reported in results) | √  3.1 Participant characteristics  Table.1 |
| S13 | Data processing | Methods for processing data prior to and during analysis, including transcription, data entry, data management and security, verification of data integrity, data coding, and anonymization/deidentification of excerpts | √  2.6 Data analysis |
| S14 | Data analysis | Process by which inferences, themes, etc., were identified and developed, including the researchers involved in data analysis; usually references a specific paradigm or approach; rationale b | √  2.6 Data analysis  Thematic analysis  Braun & Clarke,2006 |
| S15 | Techniques to enhance trustworthiness | Techniques to enhance trustworthiness and credibility of data analysis (e.g., member checking, audit trail, triangulation); rationale^b^ | √  2.6 Data analysis |
|  | **Results/findings** |  |  |
| S16 | Synthesis and interpretation | Main findings (e.g., interpretations, inferences, and themes); might include development of a theory or model, or integration with prior research or theory | √  3.2 Theme  Table.2; Figure1 |
| S17 | Links to empirical data | Evidence (e.g., quotes, field notes, text excerpts, photographs) to substantiate analytic findings | √  3 Results |
|  | **Discussion** |  |  |
| S18 | Integration with prior work, implications, transferability, and contribution(s) to the field | Short summary of main findings; explanation of how findings and conclusions connect to, support, elaborate on, or challenge conclusions of earlier scholarship; discussion of scope of application/ generalizability; identification of unique contribution(s) to scholarship in a discipline or field | √ |
| S19 | Limitations | Trustworthiness and limitations of findings | √ |
|  | **Other** |  |  |
| S20 | Conflicts of interest | Potential sources of influence or perceived influence on study conduct and conclusions; how these were managed | √ |
| S21 | Funding | Sources of funding and other support; role of funders in data collection, interpretation, and reporting | √ |

^b^The rationale should briefly discuss the justification for choosing that theory, approach, method, or technique rather than other options available, the assumptions and limitations implicit in those choices, and how those choices influence study conclusions and transferability. As appropriate, the rationale for several items might be discussed together.
